# The L1624Q Variant in *SCN1A* Causes Familial Epilepsy Through a Mixed Gain and Loss of Channel Function

**DOI:** 10.3389/fphar.2021.788192

**Published:** 2021-12-02

**Authors:** Laura B. Jones, Colin H. Peters, Richard E. Rosch, Maxine Owers, Elaine Hughes, Deb K. Pal, Peter C. Ruben

**Affiliations:** ^1^ Department of Biomedical Physiology and Kinesiology, Simon Fraser University, Burnaby, BC, Canada; ^2^ Department of Physiology and Biophysics, University of Colorado Anschutz Medical Campus, Aurora, CO, United States; ^3^ MRC Centre for Neurodevelopmental Disorders, King’s College London, London, United Kingdom; ^4^ Department of Paediatric Neurology, Great Ormond Street Hospital for Children NHS Foundation Trust, London, United Kingdom; ^5^ Department of Paediatric Neurosciences, King’s College Hospital, London, United Kingdom; ^6^ Department of Paediatric Neurosciences, Evelina London Children’s Hospital, London, United Kingdom; ^7^ Department of Basic and Clinical Neuroscience, Institute of Psychiatry, Psychology, and Neuroscience, King’s College London, London, United Kingdom

**Keywords:** childhood epilepsy, sodium channelopathies, *SCN1A* variant, gain of function, loss of function

## Abstract

Variants of the *SCN1A* gene encoding the neuronal voltage-gated sodium channel Na_V_1.1 cause over 85% of all cases of Dravet syndrome, a severe and often pharmacoresistent epileptic encephalopathy with mostly infantile onset. But with the increased availability of genetic testing for patients with epilepsy, variants in *SCN1A* have now also been described in a range of other epilepsy phenotypes. The vast majority of these epilepsy-associated variants are *de novo*, and most are either nonsense variants that truncate the channel or missense variants that are presumed to cause loss of channel function. However, biophysical analysis has revealed a significant subset of missense mutations that result in increased excitability, further complicating approaches to precision pharmacotherapy for patients with *SCN1A* variants and epilepsy. We describe clinical and biophysical data of a familial *SCN1A* variant encoding the Na_V_1.1 L1624Q mutant. This substitution is located on the extracellular linker between S3 and S4 of Domain IV of Na_V_1.1 and is a rare case of a familial *SCN1A* variant causing an autosomal dominant frontal lobe epilepsy. We expressed wild-type (WT) and L1642Q channels in CHO cells. Using patch-clamp to characterize channel properties at several temperatures, we show that the L1624Q variant increases persistent current, accelerates fast inactivation onset and decreases current density. While *SCN1A*-associated epilepsy is typically considered a loss-of-function disease, our results put L1624Q into a growing set of mixed gain and loss-of-function variants in *SCN1A* responsible for epilepsy.

## Introduction

**FIGURE 1:**
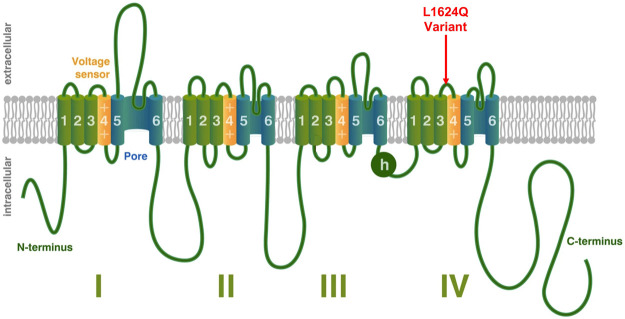
Voltage-Gated Sodium Channel Structure. Structural depiction of a mammalian voltage-gated sodium channel, modified from (Peters et al., 2016). The location of the L1624Q variant is shown with a red arrow. Other emphasized regions include the S4 voltage-sensing segments (yellow), the pore-forming regions (blue), and the intracellular loop involved in fast inactivation (h).

*SCN1A* encodes the alpha subunit of the neuronal voltage gated sodium channel Na_V_1.1 and variants of this gene are known to cause a variety of epilepsy phenotypes in humans. The alpha subunit is the primary component of the sodium channel and is sufficient to conduct current although it is typically paired with auxiliary beta subunits *in vivo* (Marban et al., 1998). Voltage gated sodium channels are comprised of four homologous domains (DI-DIV) (Noda et al., 1984). Each domain is made up of six transmembrane segments (S1–S6); S1–S4 make up the voltage sensing region, while S5 and S6 form the channel pore. Upon depolarization, the movement of the positively charged S4 segment toward the extracellular surface causes a conformational change that opens the pore, resulting in an influx of sodium (Kontis et al., 1997; Chanda and Bezanilla, 2002; Peters and Ruben, 2014). Within a few milliseconds, the intracellular loop connecting DIII and DIV (illustrated as h in Figure 1), moves to block the channel pore and prevent further sodium influx in a process termed fast inactivation (Marban et al., 1998).

The association between *SCN1A* and epilepsy is best documented for Dravet syndrome, a severe developmental and epileptic encephalopathy (Dravet, 1978; Covanis, 2012; McTague et al., 2016), with up to 85% of patients with Dravet syndrome carrying a pathogenic variant in *SCN1A* (Rosander and Hallböök, 2015; Cooper et al., 2016). Dravet syndrome presents with a difficult to treat (Chiron, 2011) epilepsy with multiple seizure types, including hemiclonic, myoclonic and tonic-clonic seizures in previously healthy infants within the first year of life, and is typically followed by developmental delays (Yakoub et al., 1992; Engel, 2001; Wolff et al., 2006; Dravet, 2011; Rosander and Hallböök, 2015). In many cases, seizures are triggered by elevated body temperature, which can be the result of a fever, exercise, or taking a hot bath (Dravet, 2011).

However, *SCN1A* gene variants are also a recognized cause for epilepsies and neurodevelopmental disorders outside the Dravet syndrome spectrum—ranging from milder phenotypes such as generalized epilepsy with febrile seizures plus (Escayg et al., 2000), to other epilepsy syndromes such as myoclonic astatic epilepsy, epilepsy of infancy with migrating focal seizures, or even to recently described more severe early onset epileptic encephalopathies with neurodegeneration and dyskinesia (Sadleir et al., 2017; Beck et al., 2019; Gorman et al., 2021). With increased genetic testing, *SCN1A* variants have been described in patients with focal epilepsies (Vezyroglou et al., 2020)—both in patients that were candidates for epilepsy surgery, and those with self-limiting focal epilepsies of childhood. These patients are part of a minority of cases of focal epilepsy for which single gene causes have been identified. Other well-characterized focal epilepsies with single gene causes include familial focal epilepsy with variable foci caused by *DEPDC5* variants (Dibbens et al., 2013), childhood epilepsy with centro-temporal spikes caused by *GRIN2A* variants (Lemke et al., 2013), and autosomal dominant nocturnal frontal lobe epilepsy (ADNFLE) associated with *CHRNA4*, *CHRNB2*, *CHRNA2*, *KCNT1*, *DEPDC5*, or *CRH* (Kurahashi and Hirose, 2002).

In a meta-analysis of genome-wide association studies (GWAS) across unselected epilepsies, *SCN1A* is one of only few genes with a significant association with epilepsy. Furthermore, other neurological and neurodevelopmental disorders, such as hemiplegic migraine and autism spectrum disorders, have now been described in the context of *SCN1A* variants (Scheffer and Nabbout, 2019). Taken together, these insights suggest much greater relevance of variants in *SCN1A* in a range of neurodevelopmental disorders extending beyond Dravet syndrome.

Over 1,200 *SCN1A* distinct variants have been associated with epilepsy (Meng et al., 2015; Gonsales et al., 2019). Although traditionally considered a loss-of-function disease, biophysical analysis has revealed a wide range of presentations, with some variants resulting in an increase in channel function, others a decrease, and some a mixture of loss- and gain-of-function (Sugawara et al., 2003; Rhodes et al., 2004; Ohmori et al., 2006; Escayg and Goldin, 2010; Liao et al., 2010; Meng et al., 2015; Peters et al., 2016; Berecki et al., 2019). This heterogeneity makes *SCN1A* epilepsies extremely challenging to treat, as a medication that improves seizures in one patient may exacerbate them in another. For example, both lamotrigine and carbamazepine have been found to worsen seizures in Dravet Syndrome (Wakai et al., 1996; Guerrini et al., 1998; Shi et al., 2016) but there has been contrasting evidence suggesting that they have been an effective therapy for a subset of patients (Dalic et al., 2015; Snoeijen-Schouwenaars et al., 2015). This highlights the importance of individualized therapy for patients with *SCN1A* related epilepsies, and the need for a better understanding of the range of epilepsy-causing variants. Unfortunately, to date the ability to consistently predict links between genotype, channel function, and phenotype remains elusive (Brunklaus and Lal, 2020).

This paper presents both clinical and biophysical data for the *SCN1A* variant c.4871T > G; p. L1624Q. In contrast to the more typical *de novo* variants, L1624Q was found across a two-generation pedigree. Unusually, affected members showed nocturnal hypermotor seizures that were confirmed to be of frontal lobe origin by video-EEG-monitoring in the index case, diagnostic of autosomal dominant nocturnal frontal lobe epilepsy without a family history of febrile seizures. This variant is located on the extracellular linker between S3 and S4 of Domain IV of Na_V_1.1 (Figure 1). Using patch-clamp to characterize channel properties, we show that the L1624Q variant causes an increase in persistent current, accelerates fast inactivation onset, and decreases current density. Experiments were conducted at both 22°C and 37°C to provide a measure of temperature sensitivity with data that allows comparison to previous studies. While most *SCN1A*-related epilepsies are typically considered a loss-of-function disease, our results put L1624Q into a growing set of mixed gain and loss-of-function variants in *SCN1A* responsible for early childhood epilepsy.

## Methods

### Patient Selection

Patients who have tested positive for missense variants in *SCN1A* on clinical genetic testing at the tertiary epilepsy service at King’s College Hospital/Evelina London Children’s Hospital, London were collated in a research database. There, an anonymized, limited set of clinical information is being captured, including syndromic electroclinical diagnoses, and limited information on family history. Ethical approval for the use of single gene sequencing data and anonymized clinical information has been given by the UK Health Research Authority (IRAS 229772).

Based on this database, we identified the patient carrying the L1624Q variant based on a syndromic diagnosis of autosomal nocturnal frontal lobe epilepsy, classically characterized by recurrent, nocturnal focal seizures of frontal lobe origin with a positive family history of similar seizures in first degree relatives suggesting an autosomal inheritance.

### Clinical Features

The identified patient was born by elective caesarean at term and there were no significant immediate problems. Her mother was on lamotrigine, sodium valproate, and carbamazepine during the pregnancy. There were some early developmental concerns with mild motor delay (walking at 18 months) and she was later identified as being hypermobile. She had delayed speech milestones, not putting sentences together until age three and half years. A comprehensive assessment at age 7.9 years demonstrated a marked discrepancy between verbal and non-verbal scores with non-verbal reasoning, visuospatial skills, and processing in the upper end of the ability range [PIQ 131 (98%)]. Verbal abilities and working memory were markedly lower especially for comprehension tasks with some scores in the impaired range so that a meaningful overall verbal IQ score could not be expressed. Further language assessments demonstrated that processing and producing language was effortful and her profile was consistent with a developmental language disorder. There was no evidence for an additional autism spectrum disorder. Of note at the time of this assessment, she was on monotherapy with low dose lamotrigine so it is unlikely that there were adverse effects of medication contributing to this profile.

The child’s habitual seizures had begun around a year of age with recurrent brief nocturnal motor seizures with the appearance on home video recordings of asymmetric predominantly tonic motor seizures. On occasions as she got older, she reported daytime visual phenomena with visual hallucinations then numbness of either arm and then headache suggestive of a migrainous etiology, though she also reported from time to time that the same visual features would occur prior to her motor seizures at night. Treatment with valproate was unsuccessful, levetiracetam made behavior worse and exacerbated the visual hallucinations, whereas lamotrigine in modest dose appeared most helpful. Higher dose appeared to make the situation worse. The addition of clobazam at night was helpful. Illness was a clear trigger for seizures, but most occurred spontaneously.

Her mother reported a history of focal seizures in several generations with her own epilepsy continuing to be difficult to control, but with lamotrigine felt to be the most helpful medication. She was cognitively able but there was an impression again of problems processing and expressing language, which appeared more than those expected as English was being spoken as a second language. She had not been formally tested. The child’s older sister had a history of epilepsy as did her older brother in his case beginning around age 6 again manifesting as recurrent nocturnal seizures. Treatment with valproate was helpful initially but seizures recurred when this was withdrawn and then did not respond to this, so levetiracetam and then lamotrigine was substituted. He also developed seizures with an initial visual aura, in his case with black and white dots and then visual obscuration evolving into predominantly asymmetric tonic seizures. He also reported that daytime seizures could be triggered by patterns. The addition of low dose clobazam at night was also helpful for him. He had additional co-morbidities with some educational difficulties, dyspraxia and autism but was in mainstream schooling throughout.

Gene profiling was performed using next generation sequencing on blood samples. No variants had been identified in the genes typically associated with autosomal dominant nocturnal frontal lobe epilepsy (CHRNA4, CHRNB2, CHRNA2 < KCNT1, DEPDC5, CRH).

### Mutagenesis and Bacterial Transformation

Wildtype *SCN1A* DNA in a PCDM8 vector was used for mutagenesis, and has been described in a previous paper (Peters et al., 2016). The L1624Q variant was generated using a QuikChange Lightening Site Directed Mutagenesis Kit (Agilent Technologies) with the following primers:

Forward: 5′ cac gaa ata ctt ttc tat ctg ctc ggc aag aaa cat acc 3′

Reverse: 5′ ggt atg ttt ctt gcc gag cag ata gaa aag tat ttc gtg 3′

DNA was transformed into TOP10/P3 *Escherichia coli* bacteria (Invitrogen) and grown at 30°C. The entire length of the sodium channel was sequenced to confirm that no spontaneous mutations had occurred.

### Cell Culture

CHOk1 cells (Sigma-Alrich) were grown in Ham’s F-12 nutrient mixture supplemented with 10% FBS (Gibco) at 37°C. Using Polyfect (Qiagen), cells were transfected with 1 μg of *SCN1A* (wildtype or mutant), 1 μg of eGFP, and 0.5 μg of the β1 subunit. Cells were plated on sterile glass coverslips approximately 24 h after transfection, and experiments were performed 24–48 h later.

### Patch Clamp Experiments

Whole cell patch clamp experiments were performed at 22°C and 37°C with an EPC9 patch-clamp amplifier (HEKA Elektronik) and Patchmaster software (Heka Electronic). Temperature was maintained with a TC-10 Temperature controller (Dagan). Glass pipettes were pulled with a P-1000 puller (Sutter Instruments), dipped in wax, and polished to a resistance of 1.0–1.5 MΩ. Intracellular solution contained 130 mM CsF, 10 mM NaCl, 10 mM EGTA, and 10 mM HEPES. Extracellular solution contained 140 mM NaCl, 4 mM KCl, 2 mM CaCl_2_, 1 mM MgCl_2_, and 10 mM HEPES. Both solutions were titrated to a pH 7.4 with CsOH.

### Protocols

To determine the voltage-dependence of channel activation, membrane potential was depolarized to potentials between −100 mV and +70 mV for 10 ms from a holding potential of −130 mV. Conductance was determined by dividing peak current by the observed reversal potential subtracted from membrane potential. Normalized conductance was plotted against voltage and fit by a Boltzmann equation. The time constant of fast inactivation onset at voltages between −30 mV and +20 mV was determined by fitting the decay of the current with a single exponential equation. Current density was calculated with current at 0 mV.

To measure the voltage-dependence of channel fast inactivation, the membrane was held at potentials ranging from −130 mV to +10 mV for 200 ms, followed by a 19 ms test pulse at 0 mV. Steady-state fast inactivation was measured as the proportion of current remaining in the 0 mV test pulse. The normalized current was plotted against voltage and fit by a single Boltzmann equation.

The rate of inactivation recovery was determined by holding the membrane potential at 0 mV for 200 ms followed by a −90 mV recovery pulse for increasing amounts of time. The time of fast inactivation recovery was measured as the proportion of current after the −90 mV recovery pulse. The normalized current was plotted versus recovery time and fit with a double exponential equation.

The fraction of non-inactivating current was measured during a 50 ms pulse from −130 to 0 mV. The percentage of persistent current was determined by dividing the current amplitude measured at the end of the 50 ms depolarizing pulse by the peak current. The current amplitude measured between 25 and 30 ms was also divided by the peak current to confirm that the persistent current had stabilized and reached steady-state.

### Statistical Analysis

Statistical analysis was performed with JMP software (SAS Institute) using a multi-factor analysis of variance. To test whether the L1624Q variant and wildtype channels have differential temperature sensitivity, the interaction between temperature and mutant was used as a predictor variable in the model. If no differential temperature sensitivity was found, temperature and mutant effects were evaluated independently. Fast inactivation onset time constants ranging from −30 mV to +20 mV were log transformed prior to analysis such that values were normally distributed. Statistical significance was defined as *p* < 0.05.

### Neuronal Action Potential Modelling

To simulate how L1624Q-dependent perturbations on channel gating alter cortical neuron firing, we modified prior models of pyramidal neuron firing (Traub et al., 1991; Destexhe et al., 1994). The methodology is described in prior publications (Peters et al., 2016; Gorman et al., 2021) and codes to run cortical neuron simulations in Python are freely available online (https://github.com/roschkoenig/SodMod). The increased rate of inactivation onset in the L1624Q variant at 37°C was implemented by multiplying the rate of inactivation onset by 1.485, reflecting the average increase in inactivation rates measured between −30 and +20 mV at 37°C. Because persistent sodium currents were not included in the original cortical neuron model, a non-inactivating (lacking the “h” gate) component of the fast sodium current was implemented with activation and deactivation rates 1,000 times those of the fast sodium current. The maximal conductance of the non-inactivating current was set at 10% of the fast component multiplied by the measured fraction of inactivating current at 37°C (1.8% in WT and 4.6% in L1624Q; see https://github.com/roschkoenig/SodMod).

We implemented single-compartment neuron models that were stimulated with three different regimes. Injections of 1–100 pA for 1 s were used to measure the effects of the L1624Q variant on the rates of action potential firing at steady-state (Figures 5A,B). To measure the effects of L1624Q on persistent action potential firing, cells were injected with 10 pA of current for 50 ms after which the stimulus was removed (Figure 5C). Finally, to measure membrane dynamics across a range of injected currents, action potential firing was measured during a slowly increasing ramp of current injection between ∼0.002 and 400 pA (Figure 6). Ramps were delivered following a period of inactivity with no current injection or following a 10 ms pre-stimulus of 10 pA to induce neuronal firing.

## Results

### L1624Q Decreases Current Density at 37°C, but Does not Shift the Voltage-Dependence of Activation

**FIGURE 2:**
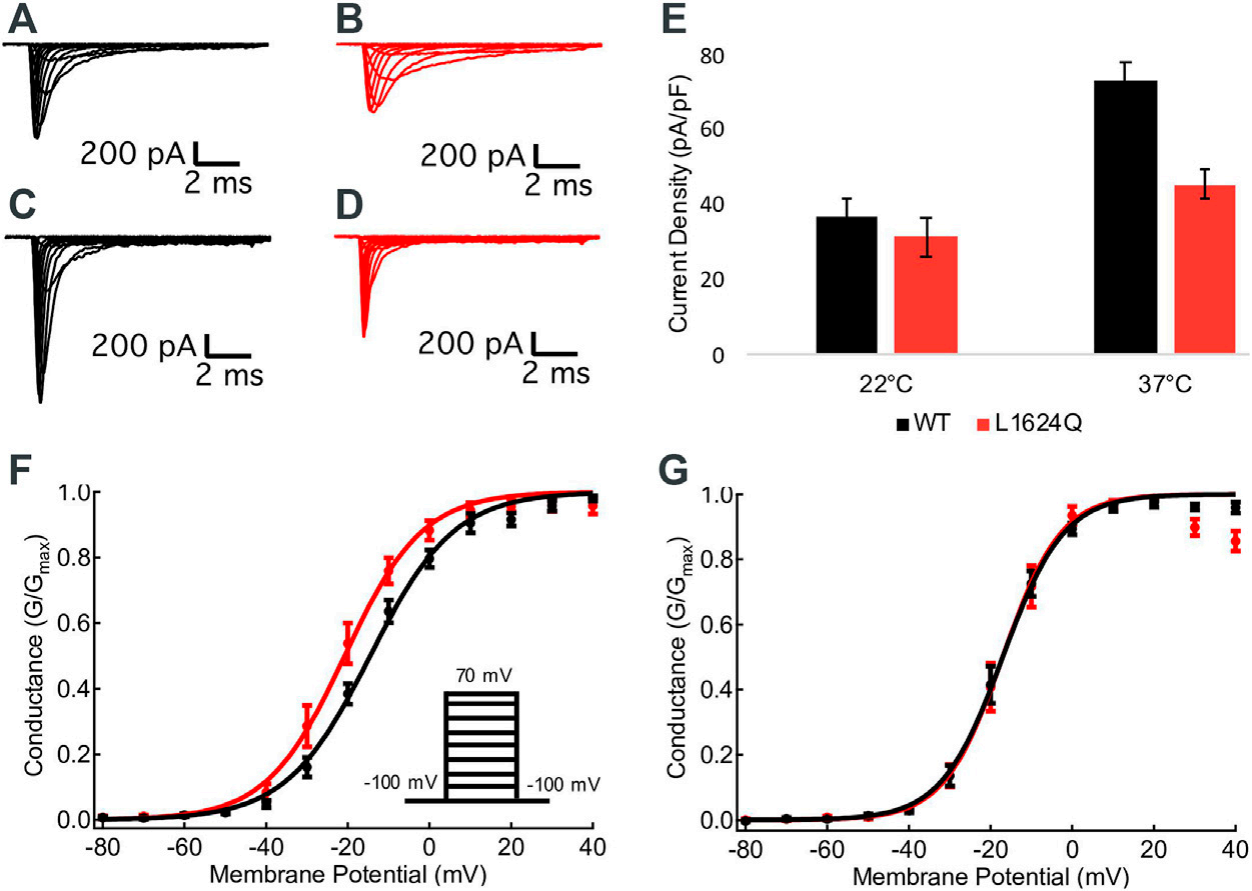
L1624Q reduces peak current density. **(A–D)**: Representative macroscopic inward sodium currents. WT (black) and L1624Q (red) traces are shown at 22°C **(A,B)** and 37°C **(C,D)**. **(E)**: Bar chart comparing mean sodium current density at 0 mV in WT and L1624Q cells at 22°C (n_WT_ = 28, n_LQ_ = 28) and 37°C (n_WT_ = 28, n_LQ_ = 41). **(F,G)**: Normalized conductance curves for WT (black) and L1624Q (red) channels are shown at 22°C (**(F)**, n_WT_ = 7, n_LQ_ = 6) and 37°C (**(G)**, n_WT_ = 10, n_LQ_ = 8). Insets show the voltage protocol. All midpoint measurements are means and error bars represent standard error of the mean.

Sample macroscopic sodium currents from WT (black) and L1624Q (red) channels at 22°C and 37°C are shown in Figures 2A–D. At 22°C, there are no significant differences in the current density between mutant and WT channels, indicating functional channel expression with the L1624Q variant (*p* = 0.5725; Figure 2E); however, current density is significantly reduced in L1624Q channels at 37°C (WT = 72.82 pA/pF, LQ = 45.42 pA/pF; *p* = 0.0021; Figure 2E). There is no significant mutant or temperature-dependent effect on the midpoint (V_1/2_) of the conductance-voltage relationship (*p* = 0.0791 and *p* = 0.4971 respectively). These results indicate that at physiological temperatures the L1624Q variant causes a loss-of-function by reducing peak current amplitudes, but this effect is not due to changes in channel activation.

### L1624Q Speeds the Rate of Fast Inactivation Onset

**FIGURE 3:**
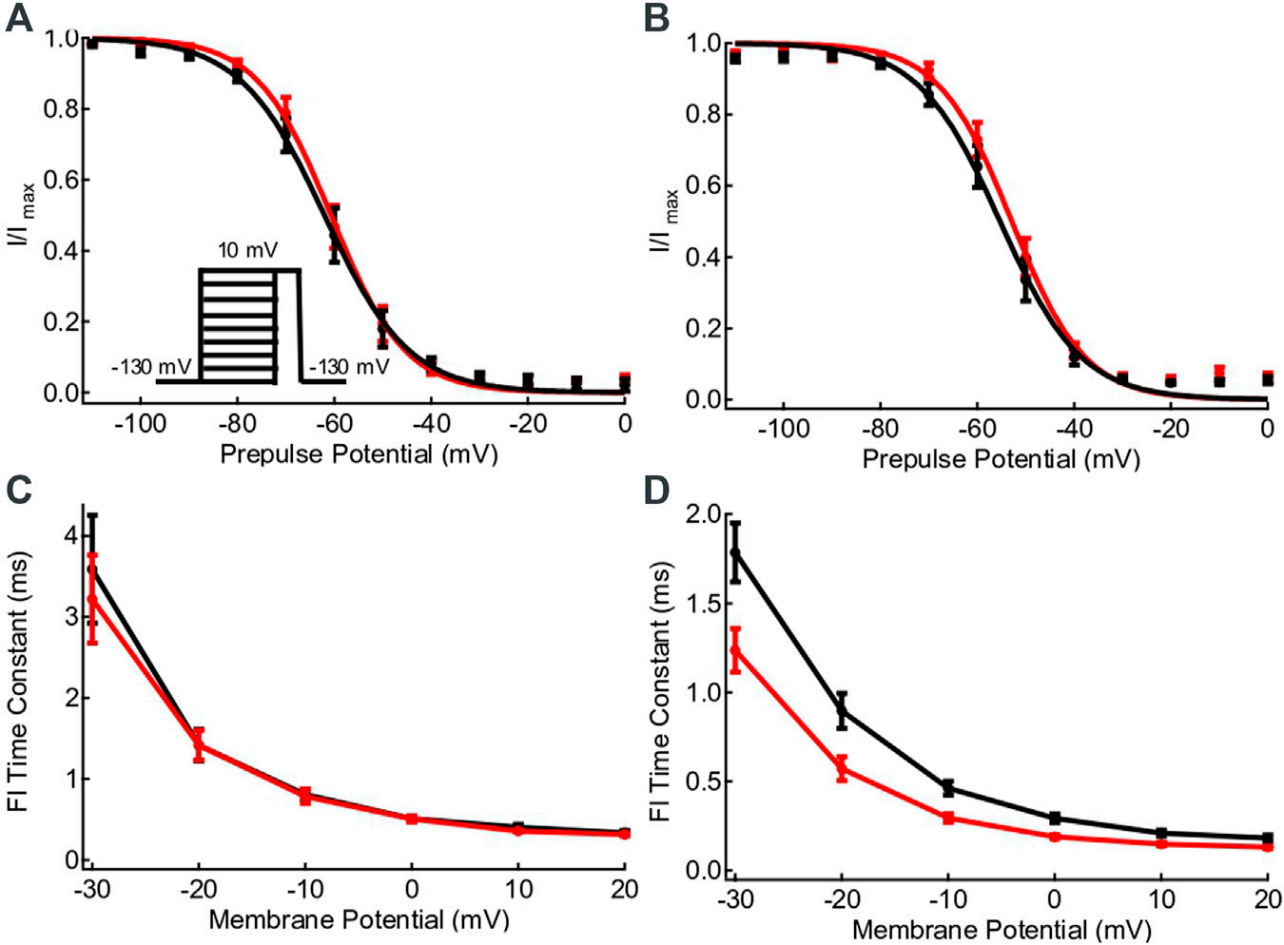
L1624Q speeds the rate of fast inactivation onset. **(A,B)**: Voltage-dependence of steady-state fast inactivation in WT (black) and L1624Q (red) channels at 22°C (**A**, n_WT_ = 9, n_LQ_ = 9) and 37°C (**(B)**, n_WT_ = 8, n_LQ_ = 8). The inset shows the voltage-protocol used to measure steady-state inactivation. **(C,D)**: Fast inactivation onset time constants plotted against membrane potential at 22°C (**C**, n_WT_ = 5, n_LQ_ = 8) and 37°C (**(D)**, n_WT_ = 7, n_LQ_ = 8). All midpoint measurements are mean and error bars represent standard error of the mean.

Steady state fast inactivation curves at 22°C and 37°C are compared in Figures 3A,B. There is a significant right shift in the midpoint of steady-state fast inactivation with increased temperature (*p* = 0.0123), but it does not differ between WT and variant (*p* = 0.6998). The rates of fast inactivation onset in the L1624Q variant are significantly more sensitive to increases in temperature compared to WT channels (*p* = 0.0089). While there are no differences in the rate of fast inactivation at 22°C in L1624Q compared to WT (*p* ≥ 0.2680; Figure 2C), the L1624Q variant increases the rate of fast inactivation onset at 37°C between −20 and +20 mV (*p* ≤ 0.0116; Figure 3D). The rate of recovery from fast inactivation at −90 mV is not significantly difference between L1624Q and wildtype (*p* = 0.8490).

### L1624Q Increases Persistent Current

**FIGURE 4:**
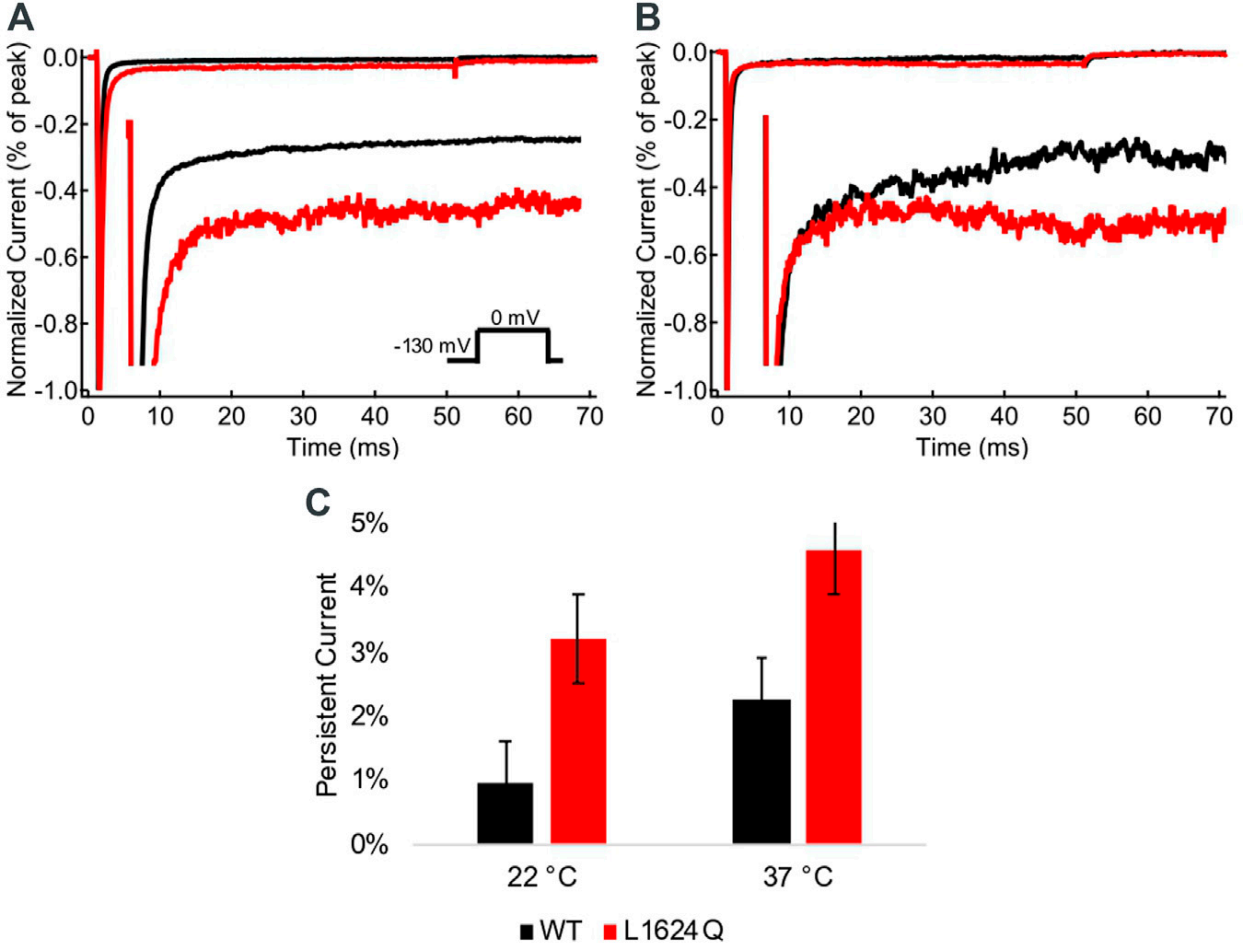
L1624Q increases persistent current. **(A,B)**: Representative normalized current traces from wildtype (black) and L1624Q (red) channels at 22°C **(A)** and 37°C **(B)**. *Insets*: Zoomed in view of the non-inactivating currents. **(C)**: Bar chart comparing the fraction of persistent current in WT (black) and L1624Q (red) channels at 22°C (n_WT_ = 5, n_LQ_ = 4) and 37°C (n_WT_ = 4, n_LQ_ = 5). All midpoint measurements are mean and error bars represent standard error of the mean.

The fraction of non-inactivating (persistent) current was measured at the end of 50 ms depolarizations at 22°C and 37°C (Figures 4A,B). Persistent current is significantly higher in L1624Q channels compared to wildtype at both 22°C (INAP_LQ_ = 3.20%, INAP_WT_ = 0.95%; *p* = 0.0039) and 37°C (Figure 4C; INAP_LQ_ = 4.58%, INAP_WT_ = 2.27%; *p* = 0.0032). In contrast to the loss-of-function in peak current amplitude, this indicates that the L1624Q variant causes a gain-of-function in the fraction of non-inactivating current.

### Neuronal Simulations

**FIGURE 5:**
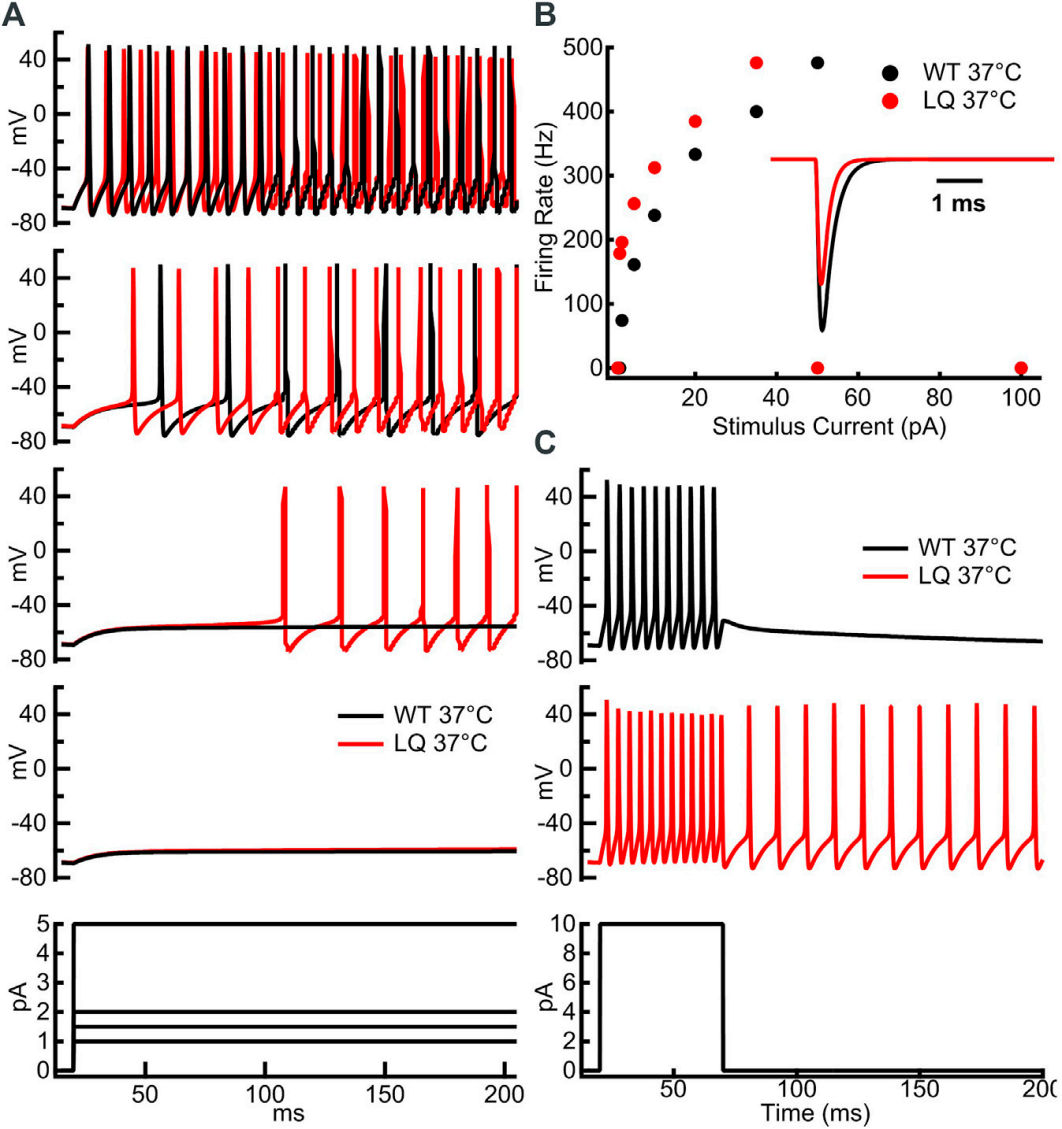
L1624Q increases neuronal excitability at lower input stimuli. **(A)**: Simulated action potential firing in WT (black) and L1624Q (red) expressing neurons at 37°C in response to stimulus currents **(bottom)** of 1 pA (second from bottom), 1.5 pA **(middle)**, 2 pA (second from top), or 5 pA **(top)**. **(B)**: Simulated action potential firing rates of WT and L1624Q expressing neurons in response to stimuli between 1 and 100 pA. **(B)** *inset*: Simulated WT (black) and L1624Q (red) sodium currents in response to a test pulse to 0 mV from a holding potential of −120 mV. **(C)**: Simulated action potential firing of WT (black) and L1624Q (red) expressing neurons in response to a 10 pA stimulus for 50 ms followed by zero stimulus **(bottom)**.

**FIGURE 6:**
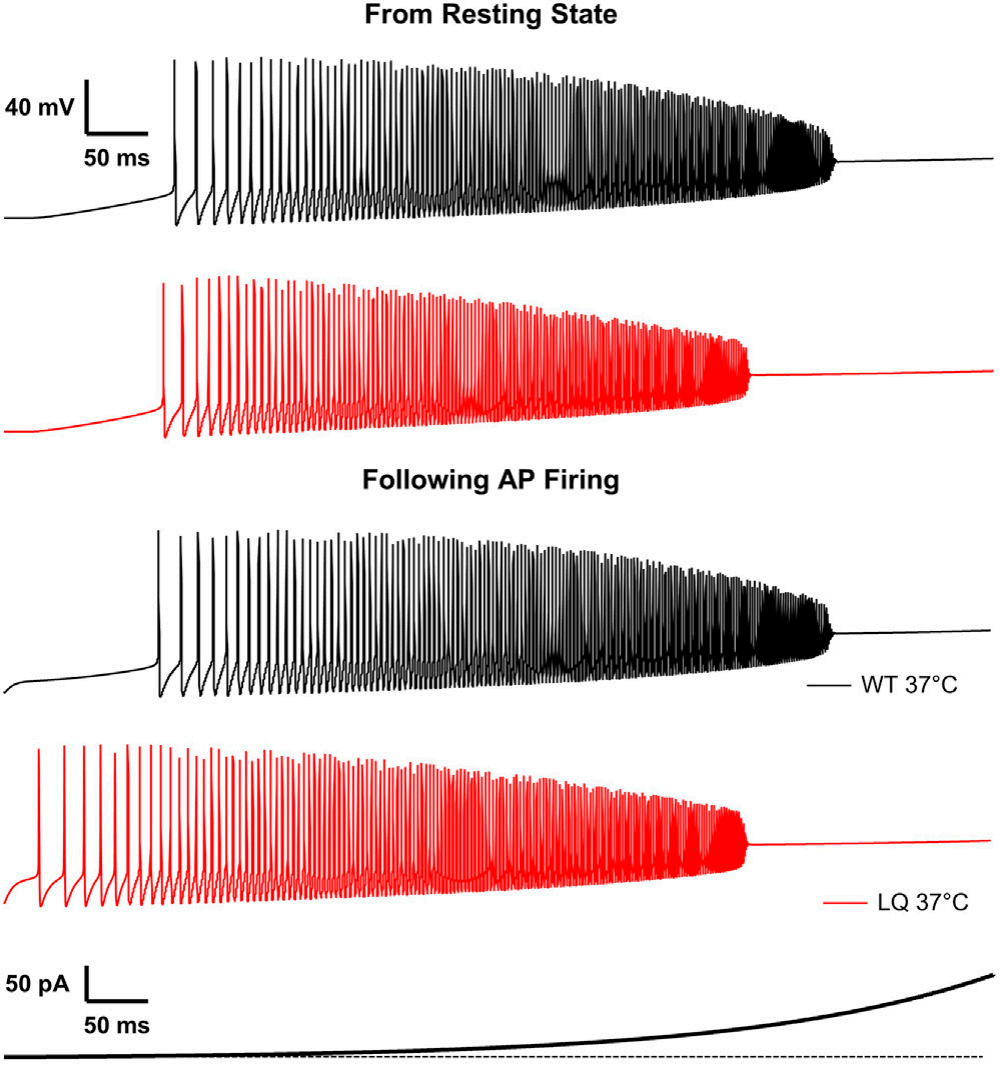
L1624Q leads to depolarization block at lower input stimuli. Simulated action potential firing in WT (black) and L1624Q (red) expressing neurons at 37°C in response to a slowly increasing stimulus current **(bottom)**. Simulations are conducted following a period of rest with zero stimulus **(top)** or following a 10 ms pre-stimulus of 10 pA to induce AP firing **(bottom)**.

To investigate the effects of the L1624Q variant on cortical neuron excitability we implemented a single-compartment pyramidal neuron simulation based on previously published models (Traub et al., 1991; Destexhe et al., 1994; Peters et al., 2016). Sodium currents in the simulated L1624Q neurons were parameterized to reflect the increased rate of fast inactivation onset and the increased fraction of non-inactivating sodium current. The increased rate of inactivation in turn decreases peak currents in the L1624Q model by approximately 25% (Figure 5B *inset*). This decrease is within the 95% confidence interval for the experimental decrease in peak current density in the L1624Q mutant (6.61−68.64%). In response to prolonged stimulations at set input currents up to 40 pA, the L1624Q expressing neurons fired at lower current injections (Figure 5A) and at slightly faster rates (Figure 5B) than WT neurons. In response to a short stimulus injection, the L1624Q simulations display persistent action potential firing that continues after the stimulus is removed (Figure 5C). Persistent firing did not terminate within the 1s simulation time. Persistent firing in the L1624Q variant is seen during a slowly increasing stimulus current ramp following a period of action potential firing (Figure 6). Interestingly, the L1624Q simulations enter depolarization block at lower stimulus currents than the WT simulations during stimulus ramps from rest or following action potential firing (Figure 6).

To further investigate the effects of increased persistent currents and faster rates of inactivation onset in L1624Q, we also modelled these effects separately. These models show that the increased persistent currents in L1624Q are primarily responsible for lower firing thresholds (Supplementary Figure S1A) and persistent AP firing (Supplementary Figure S1B). In addition, reduced current due to faster inactivation allows for slightly faster firing rates and is also partially responsible for the increased depolarization block in L1624Q (Supplementary Figure S1C). Overall, these simulations suggest that the mixed gain- and loss-of-function in the L1624Q variant leads to hyperexcitability at lower stimulus inputs and diminished excitability during larger stimuli.

## Discussion

Despite the strong association between variants of the *SCN1A* gene and Dravet syndrome, there is considerable heterogeneity in the phenotypes of *SCN1A* associated epilepsies (Sugawara et al., 2003; Ohmori et al., 2006; Escayg and Goldin, 2010; Liao et al., 2010; Meng et al., 2015; Peters et al., 2016). In fact, even in genome-wide analyses of common epilepsies, *SCN1A* is identified as a key risk gene (Anney et al., 2014) suggesting that the phenotypes associated with *SCN1A* variants extend far beyond the patients with more severe childhood onset epilepsies in whom a clinical diagnosis of *SCN1A* variants is now made on routine clinical testing. We currently lack the ability to make *in silico* predictions about channel function from genetic data (Brunklaus and Lal, 2020), limiting the current use of genetic insights in guiding treatment and prognosis for the heterogeneous *SCN1A*-related epilepsies. Understanding the functional effects of unique mutants, particularly when associated with unusual phenotypes, is an essential next step for understanding genotype-phenotype relationships in *SCN1A*-associated epilepsies. Here we show that the familial L1624Q variant is associated with increased persistent current as well as accelerated fast inactivation onset and decreased current density in response to increased temperature. Simulations of cortical neuron firing predict that this mixture of loss- and gain-of-function effects will cause both hyperexcitability, in the form of persistent firing, as well as increased entry into depolarization block. Although most *SCN1A*-associated epilepsies are considered a loss-of-function disease, our results put L1624Q into a growing set of mixed gain and loss-of-function variants in SCN1A responsible for an increasing set of epilepsy phenotypes (Meng et al., 2015).

### Familial Variants in Epilepsy

Even though epilepsy is highly heritable, only for a minority of patients can a single gene diagnosis currently be established. The yield of genetic diagnoses is highest in the early epileptic encephalopathies, for epilepsies associated with certain comorbidities, and for certain epilepsy syndromes, such as familial focal epilepsies (Mullen et al., 2013; McTague et al., 2016; Stefanski et al., 2021). However, despite the high heritability in the epilepsies generally, few patients with a familial epilepsy are diagnosed with a single causative gene mutation. In a population based Scottish national cohort study of 333 patients with epilepsy presenting with seizure onset before the age of 36 months, 15 of 80 single gene diagnoses made were in familial epilepsies with variants inherited from an affected parent (Symonds et al., 2019).

Among the familial focal epilepsies, autosomal dominant nocturnal frontal lobe epilepsy (or autosomal dominant sleep-related hypermotor epilepsy) was one of the first epilepsy syndromes in which causative single gene mutations could be identified in patients. Yet collectively the known genes only explain approximately 10% of cases. Similarly, the yield of genetic testing across familial focal epilepsies remains below ∼12% even with panel and genomic testing approaches (Tinuper et al., 2016; Oates et al., 2018).

For *SCN1A*, familial cases have initially helped establish an association with epilepsy in the context of generalized epilepsy with febrile seizures plus (GEFS+) (Wallace et al., 2001). However, most variants are now detected in sporadic cases of Dravet syndrome as *de novo* variants. Only about 10% of *SCN1A* variants are familial, and these are typically associated with milder phenotypes (Meng et al., 2015). The case presented here represents an extension of the *SCN1A*-associated epilepsy phenotypes with the clinical features of an epilepsy syndrome classically associated with different genetic causes.

### Persistent Current

Perhaps the most striking effect of the L1624Q mutant on channel activity was the large increase in persistent current passed by the channels. Despite its relatively small amplitude, persistent current can have profound impacts on sodium channel function, such as enhancing repetitive firing, heightening depolarization in the sub- and near-threshold voltage range, and reducing the threshold for action potential firing (Rhodes et al., 2004; Stafstrom, 2007; Deng and Klyachko, 2016). This abnormal neuronal activity often results in epilepsy syndromes. For example, the *SCN1A* variant P1632S, also located on the extracellular linker between S3 and S4 of Domain IV, exhibits a similar pattern of increased persistent current and an accelerated fast inactivation fast time constant τ_1_ leading to childhood epilepsy (Rhodes et al., 2005). Indeed, our simulations predict that persistent currents in the L1624Q variant lead to continued action potential firing even after a stimulating current is removed. Interestingly, lamotrigine, a sodium channel blocker that is effective in reducing persistent current at clinically appropriate doses (Stafstrom, 2007), was reported to be effective in the L1624Q patients, despite being notorious for aggravating *SCN1A* related seizures (Guerrini et al., 1998; Chiron, 2011; Anderson et al., 2017). This effect highlights the variability in response to anti-convulsant drugs and the considerable heterogeneity between patients with *SCN1A* related epilepsies.

### Mixed-Function Variants in Epilepsy

The L1624Q variant falls into a growing class of mixed loss- and gain-of-function variants in Na_V_1.1 (Ohmori et al., 2002; Claes et al., 2003; Rhodes et al., 2004; Rhodes et al., 2005; Volkers et al., 2011; Peters et al., 2016; Sadleir et al., 2017; Berecki et al., 2019; Brunklaus and Lal, 2020; Gorman et al., 2021). These variants are characterized by multiple defects that cause opposing effects on channel activation and inactivation. In many cases this takes the form similar to what we report here where increases in persistent sodium current occur along with a decrease in the peak sodium current (Escayg et al., 2001; Lossin et al., 2002; Ohmori et al., 2002; Fujiwara et al., 2003; Rhodes et al., 2004; Spampanato et al., 2004; Rhodes et al., 2005; Volkers et al., 2011). As discussed above, increases in persistent current can produce spontaneous activity and burst firing. In contrast, other variants that shift the activation and inactivation voltage dependences can alter the threshold for AP firing or alter depolarization block at higher inputs (Peters et al., 2016; Berecki et al., 2019). The L1624Q variant studied here shows a mixture of increased persistent currents and decreased peak current density. It is likely that the decreased peak current at higher temperatures is due to the rapid fast inactivation onset in this variant that may lead to significant inactivation before channels are fully activated as was shown in other sodium channel isoforms (Peters et al., 2017; Peters et al., 2020).

The mixture of loss- and gain-of-function in other voltage-gated sodium channel isoforms is known to cause overlapping syndromes with characteristics of multiple disease states; these include mixed Brugada and long QT syndrome type 3 variants in Na_V_1.5 (Bezzina et al., 1999; Abdelsayed et al., 2015) as well as mixed periodic paralysis and myotonia variants in Na_V_1.4 (Webb and Cannon, 2008; Ghovanloo et al., 2018). Thus far, the growing list of mixed variants in Na_V_1.1 have been part of the epileptic spectrum, ranging from less severe generalized epilepsy with febrile seizures (GEFS+) to severe infantile epileptic encephalopathies (Escayg et al., 2001; Lossin et al., 2002; Peters et al., 2016; Volkers et al., 2017; Brunklaus and Lal, 2020). The mixed variant T226M has been associated with a more severe early onset, atrophy of the brain, and dyskinesia due to a lack of depolarization block (Sadleir et al., 2017; Berecki et al., 2019). We recently reported a case of a large inward persistent current causing drug resistant and fatal epilepsy with atrophy and a dyskinetic disorder (Gorman et al., 2021). Drug resistance is not uncommon in epilepsy associated with SCN1A variants, and has also been reported in many of the mixed-function variants discussed above (Claes et al., 2003; Fujiwara et al., 2003; Rhodes et al., 2004; Sadleir et al., 2017). Some of these patients have benefited from anti-epileptic drugs such as lamotrigine (Volkers et al., 2011), sodium valproate (Volkers et al., 2011; Peters et al., 2016) and levetiracetam (Volkers et al., 2011), but there is no evidence that any of these drugs are a panacea for mixed-function variants in SCN1A. The current case demonstrates that specific dysfunction in Na_V_1.1 beyond simple loss of function may be associated with phenotypes not classically associated with mutations in *SCN1A*. These expansions of the phenotypic spectrum of SCN1A mutations further complicate the already tenuous relationships between genotype and phenotype (Brunklaus and Lal, 2020). Whether additional data will delineate other loss-of-function and gain-of-function variants into clear phenotypes remains to be tested.

## Data Availability

The raw data supporting the conclusion of this article will be made available by the authors, without undue reservation.

## Supplementary Material

Supplementary Figure S1 Persistent currents in L1624Q are responsible for repetitive firing. **(A)**: Simulated action potential firing rates of modified L1624Q expressing neurons at 37°C in response to stimuli between 1 and 100 pA with only variant-dependent changes in fast inactivation (blue) or persistent current (purple) included. WT (black) and L1624Q (red) data from Figure 5 are included for comparison. **(B)**: Simulated action potential firing of modified L1624Q (fast inactivation—blue; persistent current—purple) expressing neurons at 37°C in response to a 10 pA stimulus for 50 ms followed by zero stimulus. **(C)**: Simulated action potential firing in modified L1624Q (fast inactivation—blue; persistent current—purple) expressing neurons at 37°C in response to a slowly increasing stimulus current (bottom). Simulations are conducted following a 10 ms pre-stimulus of 10 pA to induce AP firing. The points at which WT (black line) and L1624Q (red line) expressing neurons stopped firing during the same protocol (Figure 6) are shown for comparison.
