# Supplementary figures and images for: The L1624Q Variant in SCN1A Causes Familial Epilepsy Through a Mixed Gain and Loss of Channel Function
Source: Front Pharmacol. 2021 Dec 2;12:788192. doi: 10.3389/fphar.2021.788192 (PMC8675213; doi:10.3389/fphar.2021.788192)

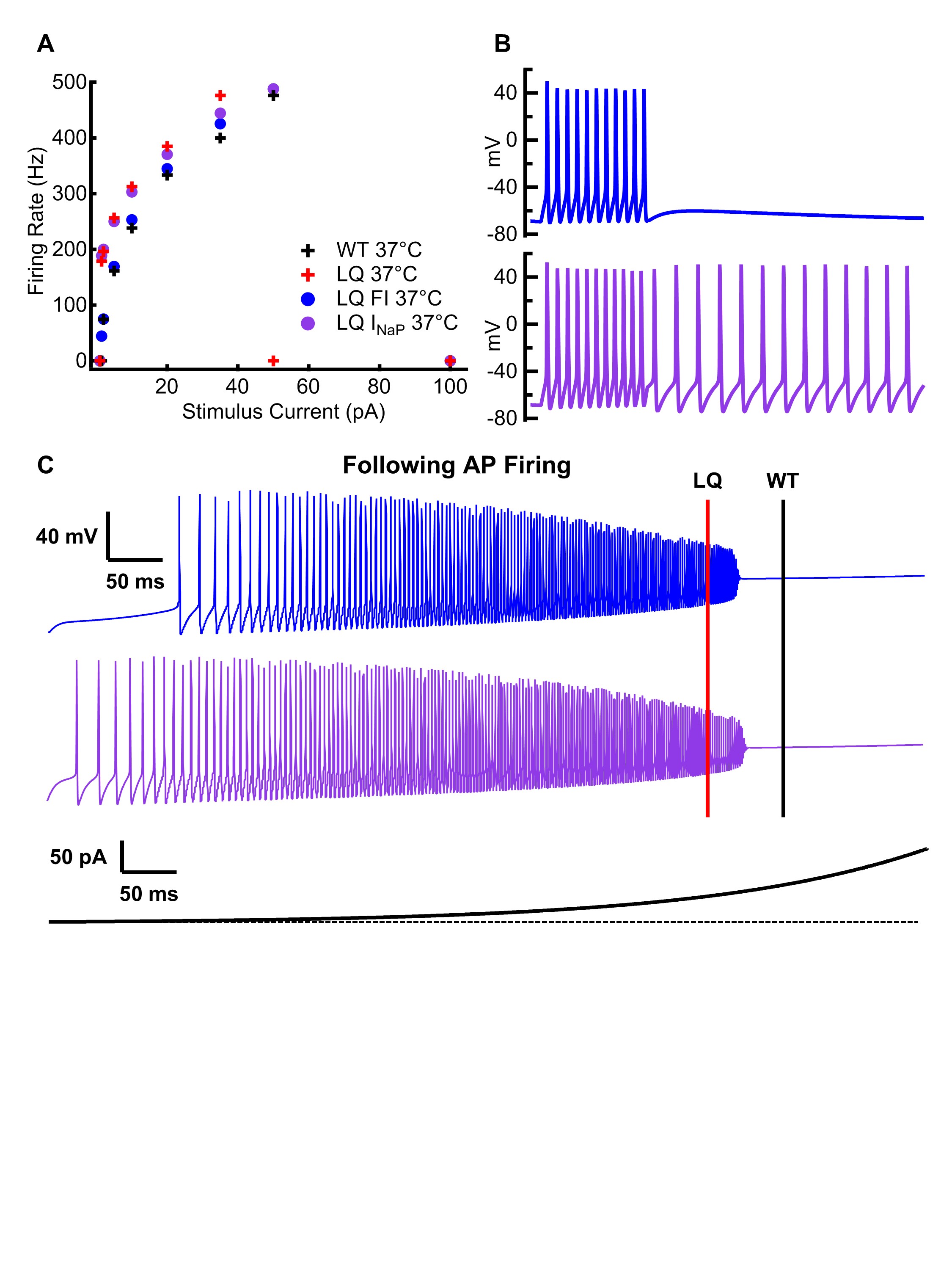

Supplement: Supplementary file 1 [file Image1.JPEG]
